# Effectiveness of electric toothbrush as vibration method on orthodontic tooth movement: a split-mouth study

**DOI:** 10.1590/2177-6709.24.2.049-055.oar

**Published:** 2019

**Authors:** Muhammad Azeem, Ambreen Afzal, Saqib Ali Jawa, Arfan Ul Haq, Mahwish Khan, Husnain Akram

**Affiliations:** 1 Faisalabad Medical University, Department of Orthodontics (Faisalabad, Pakistan).; 2 Altamash Institute of Dental Medicine, Department of Orthodontics (Karachi, Pakistan).; 3 King Khalid Hospital (Tabuk, Saudi Arabia).; 4 de’Montmorency College of Dentistry, Postgraduate Program (Lahore, Pakistan).

**Keywords:** Electric toothbrush, Orthodontic tooth movement

## Abstract

**Objective:**

To investigate the effects of application of vibratory stimuli, using an electric toothbrush, on the rate of orthodontic tooth movement during maxillary canine retraction.

**Methods:**

A split-mouth study was conducted in 28 subjects (mean age = 20.8 years; ranging from 18 to 24 years) whose bilateral maxillary first premolars were extracted with subsequent canine retraction. On the Vibration side, light force (100 g) was applied to the canine for 90 days, in combination with vibratory stimuli provided by an electric toothbrush; only orthodontic force was applied to the canine on the non-vibration side. Amount of canine movement was measured monthly. Related to electronic toothbrush usage, a diary was provided to each patient for recording discomfort during experimental period, having 100-mm visual analogue scale (VAS). The paired t-test was used to assess the differences in amount of tooth movement between canines of the vibration and non-vibration sides.

**Results:**

The amount of tooth movement was similar for canines on the vibration side and on the non-vibration side (mean 0.81 ± 0.10 mm and 0.82 ± 0.11 mm, respectively, *p*> 0.05). Plaque accumulation was minimal in any subject throughout the study. No subject reported discomfort as a result of using the electric toothbrush.

**Conclusions:**

This study demonstrates that application of vibratory stimuli using an electric toothbrush, in combination with light orthodontic force, do not accelerate orthodontic tooth movement.

## Resumo

**Objetivo:**

investigar os efeitos da aplicação de estímulo vibratório, usando escova elétrica, sobre a taxa de movimentação dentária ortodôntica durante a retração dos caninos superiores.

**Métodos:**

um estudo de boca-dividida foi realizado em 28 pacientes (idade média de 20,8 anos, variando entre 18 e 24 anos) cujos dois primeiros pré-molares superiores foram extraídos, com subsequente retração dos caninos. No lado Com Vibração, uma força leve (100g) foi aplicada no canino durante 90 dias, em combinação com o estímulo vibratório gerado por uma escova de dentes elétrica; enquanto os caninos do lado Sem Vibração foram submetidos apenas à aplicação da força ortodôntica. A quantidade de movimentação dos caninos foi aferida mensalmente. Quanto ao uso da escova de dentes elétrica, diários foram fornecidos aos pacientes para que esses anotassem, em Escalas Visuais Analógicas (EVA) de 100 mm, o desconforto sentido durante o período experimental. O teste *t* pareado foi utilizado para avaliar as diferenças na quantidade de movimentação dos caninos nos lados Com Vibração e Sem Vibração.

**Resultados:**

os valores da movimentação dentária foram semelhantes nos lados Com Vibração e Sem Vibração (médias de 0,81 ± 0,10 mm e 0,82 ± 0,11 mm, respectivamente, *p*> 0,05). O acúmulo de placa dentária nos pacientes dessa amostra foi mínimo, ao longo de todo o estudo. Nenhum paciente relatou desconforto durante o uso da escova elétrica.

**Conclusões:**

o presente estudo demonstrou que a aplicação de estímulo vibratório usando uma escova elétrica, associada a forças ortodônticas leves, não foi capaz de acelerar a movimentação dentária ortodôntica.

## INTRODUCTION

Orthodontic tooth movement is a complex process in which various molecular proteins - like receptor activator of nuclear factor kappa B (RANK), its ligand (RANKL), and osteoprotegerin (OPG) - interact to regulate the bone remodelling process.^1^ ^-^ ^3^ One of the common deterrents to orthodontic therapy is the amount of time in which a patient needs to commit, thus, there has been a continuous search for techniques to accelerate the rate of orthodontic tooth movement.^4^ At present, there are various invasive techniques to accelerate the rate of orthodontic tooth movement, namely: surgically facilitated orthodontics;^5^ combination of interradicular corticotomy and supra-apical osteotomy technique;^6^ ^,^ ^7^ periodontal ligament distraction;^8^ undermining of interseptal bone;^9^ the corticotomy-facilitated technique;^10^ dentoalveolar distraction osteogenesis;^11^ micro-osteoperforation,^12^ and piezopuncturing.^13^

Many efforts have been made to develop non-invasive methods that could speed up the rate of orthodontic tooth movement by increasing alveolar bone turnover rate. One such latest technique is Vibratory stimuli, which has the potential to speed up the rate of tooth movement. Nishimura et al^14^ found that in rats, vibrations could speed up the rate of tooth movement by stimulating the expression of RANKL and osteoclastogenesis in periodontium, and enhancing bone remodelling. Although one study in orthodontic patients stated that vibrations play no role in speeding up the rate of orthodontic tooth movement during initial alignment;^15^ in contrast, other studies reported that vibratory stimuli can accelerate the rate of tooth movement in humans by 2-3 mm/month without causing root resorption.^16^ ^,^ ^17^ Recent clinical trial revealed that vibrations using AcceleDent^®^ device at the frequency of 30 Hz is a safe and successful way of accelerating tooth movement during orthodontic treatment.^18^

Thus, the aims of the present clinical study were: to investigate the effect of applying vibratory stimuli, using an electric toothbrush, on the rate of maxillary canine retraction; to evaluate pain discomfort assessed on the VAS scale; and to evaluate plaque index. This research project is important as it added data into the orthodontic literature regarding the effects of electric toothbrush vibratory stimuli on canine retraction. 

## MATERIAL AND METHODS

This study was approved by the Ethics Committee of Orthodontic Department, de’Montmorency College of Dentistry, and Dental Section, Faisalabad Medical University, Pakistan (3074/DCD). All subjects and their parents or guardians consented to participation after receiving verbal and written explanations. Sample size was calculated by means of BioEstat v. 5.3 software, based on an internal pilot study. As per results of this pilot study, the variable amount of canine retraction per month was used. From this, sample size was determined with a test power of 80% and α = 5%, and 18 patients were required. Twenty-eight orthodontic patients (18 females, 10 males; mean age = 20.8 years; range 18 - 24 years) were randomly selected from the Orthodontic department. 

For selecting the patients, the following criteria were used: (1) need for bilateral maxillary first premolar extractions (with moderate anchorage requirements) and fixed appliance orthodontic therapy; (2) similar minimal crowding on each side of the maxillary arch; (3) no previous orthodontic therapy; (4) no past or present signs and symptoms of periodontal disease; and (5) all teeth having plaque index lower than 10%. Before bonding, all patients underwent supragingival scaling and polishing, and were given instructions on dental hygiene. They were instructed to brush and floss their teeth thrice a day. Patients were excluded if they had: A mental handicap, a physical handicap that restricted free movement of hands or fingers, craniofacial anomalies, history of recent trauma or recent oral surgery, significant medical history or medication that would adversely affect orofacial development and any subsequent tooth movement.

This study used a split-mouth design; the vibration side was randomly allocated using random number tables. After extraction of premolars, a three-month consolidation period was allowed to permit equal amounts of bone formation at the extraction sites adjacent to the canines, before the experimental period, ensuring similar quality of bone around the canine roots. Each patient was treated with MBT’s prescription preadjusted edgewise brackets (3M Gemini brackets; 3M Unitek Corporation, Monrovia, Calif) with 0.022-in slots. Initial alignment was done with a 0.014- or 0.016-in NiTi wire (3M Unitek, Monrovia, Calif), taking an average of 1.8 months (range 0.4 - 5.9 months). Then, a 0.019 × 0.025-in TMA (TMA, 3M Unitek) archwire was left *in situ* for two months to obtain standardized first-, second-, and third-order prescriptions for the experimental teeth. Segmental 0.020-in stainless-steel wire (Dentaurum, Ispringen, Germany) was passively engaged, using stainless steel ligatures, before starting canine retraction by pre-calibrated superelastic NiTi closing coil spring (GAC International, Bohemia, NY) with 100 g of force. Closed coil spring was attached from molar band hook to canine bracket hook, on both sides. No reactivation of the closing coils was needed at the start of the second and third months of retraction (labelled R2 and R3). 

After the first month of retraction (R1), the right or left canine was randomly selected (by the trial supervisor) as the vibration side tooth, for additional stimulation with an oscillating-rotating electric toothbrush with a specially designed orthodontic brush head (Oral-B Triumph, OD17; Procter & Gamble, Cincinnati, Ohio) (125 Hz). The patients were then instructed not to clean their teeth with the electric toothbrush; rather, they were instructed to hold the toothbrush to apply mechanical vibration on the mesiolabial surface of vibration side canine for a minimum of 20 minutes a day, for 60 days. The participants were requested to note and report the duration of use, and were scheduled to visit once a month, during which batteries were provided for the electric toothbrush. It was ensured that patients used the brush only for the stimulation, and not for brushing teeth. 

Plaque assessment and monitoring was done by plaque index (PI), conducted at six sites per tooth at four different periods (R0, R1, R2, and R3) after bonding, by a blinded examiner (0 = no plaque; 1 = thin film of plaque adhered to the gingival margin and adjacent area of the tooth; 2 = moderate accumulation of plaque within the gingival sulcus seen with the naked eye, or on the tooth and gingival margin; 3 = abundance of plaque within the gingival sulcus or on the tooth and gingival margin). Assessment comprised first molar, second premolars, canines and central and lateral incisors of each upper hemiarch. 

Related to electronic toothbrush usage, a diary was provided to each patient, for recording discomfort during experimental period. Discomfort was assessed on the 100-mm visual analogue scale (VAS) by asking participants to make a line across the scale, corresponding to perceived discomfort. Similarly, the analgesic consumption was recorded as “yes or no” response for each day. The VAS score in millimeters was measured from the left margin of the scale to the nearest millimeter, using a metallic ruler to quantify the discomfort. Discomfort diary data from the VAS were collected from each patient daily on the first 7 days of each month of the experimental period. These scores were averaged to determine the first week score. After the first week, discomfort was scored once weekly, for the remainder of the month. The four weekly scores were averaged to represent a monthly score, for comparisons. Participants were continuously reminded to complete their VAS in diary and record discomfort scores, and whether they were taking rescue medications. At the end of the experimental period, patients returned the discomfort scale data. 

A series of plaster models from each subject were used to assess the amount of canine retraction relative to the stable landmark of the ipsilateral median end of the third palatal rugae. Each initial model was used for making the palatal plug, with reference wires pointing at the mesial contact of the canines.^20^ The plug was then transferred to the consecutive models to measure displacement of the mesial contact of the canines relative to the reference wires. One investigator blinded to the experiment measured all models with a digital calliper (General Tools, New. York, NY) to an accuracy of 0.01 mm.

### Statistical analysis

The amount of tooth movement was measured thrice by the responsible investigator at a 4-week interval. Intraclass correlation coefficient was used to assess intraobserver reliability. In the data collected for pain and PI, Friedman test was used to detect potential differences in PI among the analyzed periods. The paired t-test was used to assess the differences in amount of tooth movement between the canines on vibration and non-vibration sides, in conjunction with 95% confidence intervals (95% CI). The Shapiro-Wilk test was used for normality. The Statistical Package for the Social Sciences (version 20.0; SPSS) was used for data analysis, with *p*<0.05 indicating statistical significance.

## RESULTS

**Figure 1:**
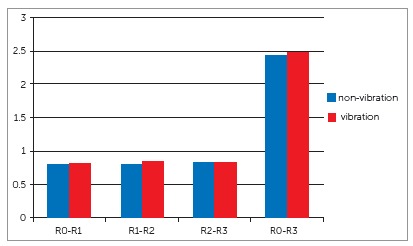
Comparison of amount of retraction (mm) of the canines on vibration and non-vibration sides.

The intraclass correlation coefficient (0.91) showed excellent reproducibility and reliability. The data was normally distributed, as tested with the Shapiro-Wilk test. During the first month of canine retraction (R0-R1), the amount of canine movement was equal for the vibration and non-vibration sides. The amount of canine movement at R2 and R3, after the first month of retraction, in combination with vibratory stimulation by electric toothbrush, was also similar for the canines on vibration and non-vibration sides (*p*> 0.05; 95% CI) (Tables 1 and 2) (Fig 1). 

**Table 1:** Mean (± standard deviation) amount of retraction (mm) of the canines on vibration and non-vibration sides.

Time	R0-R1	R1-R2	R2-R3	R0-R3
Non -vibration	0.80 ± 0.10	0.81 ± 0.11	0.82 ± 0.09	2.43 ± 0.30
Vibration	0.81 ± 0.11	0.84 ± 0.09	0.83 ± 0.13	2.48± 0.33
P value	1.000	1.000	1.000	1.000

**Table 2:** Comparison of both sides.

	t-test						
	t	Df	Sig. (2-tailed)	Mean Difference	Std. Error Difference	95% Confidence Interval of the difference	
						Lower	Upper
Amount of canine retraction mm	4.118	56	0.000	2.13300	1.03987	0.66723	0.917009

Plaque accumulation was minimal in any subject throughout the study. The PI values had no statistically significant differences between the vibration and non vibration sides (*p*> 0.05). The PI was low for all examined teeth (score 0 = 93%; score 1 = 6.82%; score 2 = 0.18%) during the whole experimental period (from R0 to R3). The PI did not change significantly from the baseline values to the end of experimental period (*p*> 0.05) (Table 3).

**Table 3:** P values from the statistical comparison of vibration and non-vibration sides.

Time	R0 (PI)	R1 (PI)	R2 (PI)	R3 (PI)
Non-vibration	1.000	1.000	1.000	1.000
Vibration	1.000	1.000	1.000	1.000

No subject reported discomfort as a result of using the electric toothbrush. A statistically nonsignificant difference in change of pain score over time was found in the experimental period (*p*> 0.05) (Table 4). No patient was noncompliant with their pain diary, and no one reported usage of rescue medications. 

**Table 4:** Mean pain scores over time*, related to electric toothbrush usage for 20min/day.

Time	R1	R2	R3
Overall pain score*	3.31	3.49	3.78

*Pain scores from the first seven days were averaged to make the first week score. After the first week, pain was scored weekly for the following three weeks. The four weekly pain scores were averaged again to represent a monthly score.

## DISCUSSION

The number of adults receiving orthodontic therapy is increasing, and the main concern for them is prolonged treatment duration, which poses high risks for caries, root resorption, and decreased patient compliance and satisfaction.^4^ ^,^ ^18^ Various techniques for accelerated tooth movement are invasive in nature as they involve surgical insult.^4^ ^-^ ^12^ Recently, an atraumatic technique of accelerated tooth movement by accelerating periodontal and alveolar bone remodelling using vibratory stimulation was introduced.^19^ ^,^ ^20^

The aim of the current study was to explore the effect of vibratory stimuli provided by an electric toothbrush on the rate of orthodontic tooth movement. It was found that the application of vibratory stimuli using an electric toothbrush 20 minutes a day was not effective in accelerating orthodontic tooth movement without causing patient discomfort. 

Vibration applied to one specific tooth could be translated to the other ones through the archwire, that’s why segmented mechanics was used as it could help in controlling this side effect. This is in agreement with previous studies that showed that vibrations could not speed up the rate of tooth movement,^15,21^ but in contrast with other studies that showed significant advantage in using the vibrational appliance for speedy orthodontics.^13,16-20,22^ A recent study using the Tooth Masseuse device in orthodontic patients reported no effect on the rate of tooth movement, which is in agreement with the present results.^22^ This is perhaps because the electronic toothbrush was never intended or designed to accelerate tooth movement, and have insignificant potential to stimulate molecular mechanisms controlling acceleratory tooth movement. 

Studies on corticotomy^23^and micro-osteoperforations^24^ revealed that these minor oral surgical procedures are effective in accelerating orthodontic tooth movement. Furthermore, a recent systematic review and meta-analysis on methods of accelerating orthodontic tooth movement, not including mechanical vibratory stimuli, found some evidence for the effectiveness of corticotomy surgical procedures.^25^ The present results, which are in contrast with studies on surgical procedures such as corticotomy^23^and micro-osteoperforations,^24^ can be linked to the higher output frequency and low force levels of these electric brushes, which in turn get ineffective in stimulating Interlukin (IL)-1b secretion by osteoclasts.^26,27^

Regional Accelerated Phenomenon^28^ at the extraction site may have affected the rate of canine movement; but, in the present study, bonding was performed 90 days after maxillary first premolar extraction, to permit equal amounts of bone formation at the extraction sites adjacent to the maxillary canines before the experimental period, ensuring similar quality of bone around the maxillary canine roots.

The present results showed statistically insignificant difference in the amount of canine retraction on vibration side, compared to the control side. These results are in contrast with Leethanakul et al,^22^ who showed that the amount of canine retraction remained increased for experimental side canine, when compared with control side. Retraction force was applied with elastomeric chain in Leethanaku’s study,^22^ but in the current study NiTi closed coil spring was used for continuous and controlled forces. The monthly rate of retraction for the canines of control and vibration sides was approximately 0.80 mm/month, which favorably compares with earlier reports.^19,29,30^ This is perhaps because the electronic toothbrush was never intended or designed to accelerate tooth movement: its output frequency is four times higher compared to other studies,^16^ ^-^ ^20^ while the force is about four times lower. Moreover, in the current study, patients were instructed to apply mechanical vibration for 20 minutes per day, in accordance with previous studies where the same time protocol was used for applying vibratory stimuli,^16^ ^-^ ^20^ ^,^ ^21^ ^,^ ^31^ while Leethanakul et al^22^ instructed patients to apply mechanical vibration for 5 minutes, three times a day, using electric toothbrush. 

No subject reported discomfort as a result of using the electric toothbrush. A statistically nonsignificant difference in change of pain score over time was found during experimental period. A VAS was used in the present study to assess discomfort. This scaled method is a reliable method of measuring discomfort, and test-retest reliability for the VAS has been shown to be very good.^32^Several studies have found that vibratory stimuli diminishes pain responses.^33^ ^-^ ^35^However, at least one study found no pain relief with the use of a vibrations.^21^

Recent research showed that the electric toothbrush, with either brush head, demonstrated significantly greater plaque removal compared to manual toothbrush.^36^ Furthermore, one study concluded that electric toothbrushes can improve plaque control without causing damage to the components of the orthodontic appliance.^37^ Most subjects in this study used the electric toothbrush as instructed, and all subjects reported that this toothbrush was comfortable and practical to use. 

This study suggests vibratory stimuli by electronic toothbrush could be a new area for further research on accelerated tooth movement, as current evidence is missing. This research project was important as it added data into the orthodontic literature regarding effects of electronic toothbrush vibratory stimuli during canine retraction. Limitations of this study are short term study duration and small sample size. Furthermore, the present study did not focus on the underlying mechanism by which vibratory stimuli accelerated canine retraction. Therefore, future studies with larger sample size and long term clinical duration are suggested. Our future studies will focus on understanding the signalling pathways associated with vibratory stimuli during canine retraction. 

## CONCLUSION

Clinical application of vibratory stimuli using an electronic toothbrush cannot be recommended with the purpose of accelerating the rate of orthodontic tooth movement.
